# Primary Immunodeficiency Disease Mimicking Pediatric Bechet’s Disease

**DOI:** 10.3390/children8020075

**Published:** 2021-01-22

**Authors:** Mayuka Shiraki, Saori Kadowaki, Tomonori Kadowaki, Norio Kawamoto, Hidenori Ohnishi

**Affiliations:** 1 Department of Pediatrics, Gifu University Graduate School of Medicine, Gifu 501-1194, Japan; cafe_flavour624@yahoo.co.jp (M.S.); saori222n@yahoo.co.jp (S.K.); fact_or_fiction_0811@yahoo.co.jp (T.K.); noriok@gifu-u.ac.jp (N.K.); 2 Department of Pediatrics, National Hospital Organization, Nagara Medical Center, Gifu 502-8558, Japan; 3 Clinical Genetics Center, Gifu University Hospital, Gifu 501-1104, Japan

**Keywords:** pediatric Bechet’s disease, primary immunodeficiency disease, autoinflammatory syndrome, NF-κB signaling pathway, TNFAIP3

## Abstract

Behcet’s disease (BD) is a chronic inflammatory disease with multisystemic involvement. Its etiology is considered to involve complex environmental and genetic factors. Several susceptibility genes for BD, such as human leukocyte antigen (HLA)-A26, *IL23R-IL12RB2*, *IL10* and *ERAP1*, in addition to the well-studied HLA-B51, were mainly identified by genome-wide association studies. A heterozygous mutation in *TNFAIP3*, which leads to A20 haploinsufficiency, was found to cause an early-onset autoinflammatory disease resembling BD in 2016. Several monogenic diseases associated with primary immunodeficiency disease and trisomy 8 have recently been reported to display BD-like phenotypes. Among the genes causing these diseases, *TNFAIP3*, *NEMO*, *RELA*, *NFKB1* and *TNFRSF1A* are involved in the NF-κB (nuclear factor κ light-chain enhancer of activated B cells) signaling pathway, indicating that this pathway plays an important role in the pathogenesis of BD. Because appropriate treatment may vary depending on the disease, analyzing the genetic background of patients with such diseases is expected to help elucidate the etiology of pediatric BD and assist with its treatment. Here, we summarize recently emerging knowledge about genetic predisposition to BD.

## 1. Introduction

Behcet’s disease (BD), as characterized by four core phenotypes (skin erythema, uveitis, genital ulcerations, and recurrent aphthous stomatitis), was initially identified in 1937 by Hulusi Behcet [1]. Diagnosis of BD is based on the published criteria for this disease, such as the criteria from the International Study Group for the diagnosis of BD (ISGBD) (1990) [2]. Some modifications to these criteria have been added over time; the most recent version of the diagnostic criteria of pediatric BD, a consensus classification criterion for pediatric BD from a prospective observation cohort (PEDBD), is now widely used [3].

The etiology of BD is considered to involve a complex interplay between environmental and genetic factors. The most extensively studied genetic factor for BD susceptibility is human leukocyte antigen (HLA)-B51 [4], but several studies including genome-wide association studies (GWAS) have identified many other BD susceptibility genes (HLA-A26, *IL23R-IL12RB2*, *IL10*, *CCR1*, *ERAP1*, *KLRC4*, *STAT4*, *MEFV*, *IL12A*, *FUT2*, *IL1A-IL1B*, *RIPK2*, *ADO-EGR2*, *LACC1*, *IRF8*, and *CEBPB-PTPN1*) [4,5,6,7,8,9,10]. However, despite the long wait, no monogenic factor has been reported that explains the etiology of BD.

However, a heterozygous loss-of-function mutation in the *TNFAIP3* gene, which causes an early-onset autoinflammatory disease resembling BD (namely, A20 haploinsufficiency; HA20), was identified at the end of 2015 by Ivona Aksentijevich’s laboratory [11]. In fact, it transpires that many patients who carry a pathogenic mutation in the *TNFAIP3* gene experience juvenile-onset BD [12,13].

Autoinflammatory diseases (AID) (including HA20) are categorized as one of many primary immunodeficiency diseases (PIDs). To date, over 400 diseases have been registered as PIDs in the International Union of Immunological Societies Expert Committee classification [14]. Not only HA20, but many other monogenic diseases also have been reported to show BD-like phenotypes. In this review, we summarize genetic predisposition to BD. As the major genetic factor for the onset of BD, we initially focus on the nuclear factor κ light-chain enhancer of activated B cells (NF-κB) pathway. Because the NF-κB pathway plays an important role in the pathogenesis of BD and BD-like phenotypes [15], we will describe the genes by dividing them into those related or not related to this pathway. Table 1 summarizes the PID-related genes and chromosomes associated with BD-like phenotypes.

## 2. NF-κB-Related Genes

**Figure 1:**
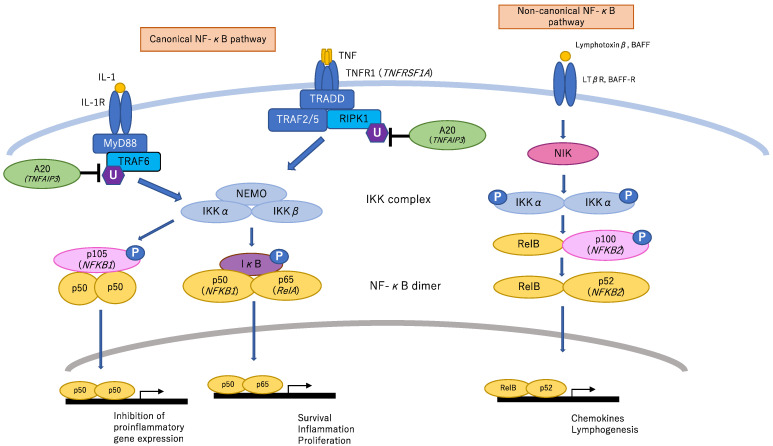
NF-κB signaling pathway and the genes associated with BD-like phenotypes. In the canonical pathway, when signals such as TNF-α and IL-1β are recognized by cytokine receptors, RIPK1 (Receptor-interacting serine/threonine-protein kinase 1) and tumor necrosis factor receptor-associated factor 6 (TRAF6) are ubiquitinated and the IκB kinase (IKK) complex is activated. Activation of the IKK complex leads to IκB phosphorylation and NF-κB (p50/p65) activation. Activated NF-κB is translocated into the nucleus where it activates targeted gene expression. P50 homodimers curb inflammatory reactions. A20 acts on ubiquitination and suppressively regulates this pathway. In particular, upon TNF receptor stimulation, A20 removes K63-linked polyubiquitin chains, thereby preventing the interaction of RIPK1 with NEMO, and subsequently adds K48-linked polyubiquitin chains to RIPK1, targeting it for proteasomal degradation. Furthermore, upon IL-1 receptor binding, A20 removes K63-linked polyubiquitin chains from TRAF6. In the non-canonical pathway, when ligands such as lymphotoxin-β and BAFF (B cell-activating factor belonging to the TNF family) bind to the receptor, NF-κB inducing kinase (NIK) is activated. Phosphorylation of IKK complex by activated NIK leads to p100 (IκB domain) phosphorylation and NF-κB (p52/RelB) activation.

NF-κB, a multi-protein complex that acts as a transcription factor, contains the following five constituent proteins: p50, p53, p65 (RelA), c-Rel, and RelB. The NF-κB signaling pathway regulates many cellular processes including cytokine production, cell proliferation and apoptosis by activating NF-κB, and is tightly regulated through multiple mechanisms. Figure 1 shows the NF-κB signaling pathway and the genes associated with BD-like phenotypes.

### 2.1. TNFAIP3

*TNFAIP3* (OMIM: 191163) encodes the A20 protein, is involved in ubiquitin modification (Figure 1), and acts suppressively on the NF-κB pathway and on interferon regulatory factor (IRF) signaling related to type I interferon (IFN) production.

HA20 (OMIM: 616744), which is caused by a loss-of-function mutation in *TNFAIP3* was identified at the end of 2015. The loss-of-function mutation in *TNFAIP3* diminishes the inhibitory effect of NF-κB, leads to increased inflammatory cytokine production and causes systemic inflammatory disease. To date, HA20 is the most common monogenic disease with BD-like phenotypes [26,27]. Patients carrying HA20 present with periodic fevers, recurrent aphthous stomatitis, genital ulceration, intestinal symptoms, skin rashes, polyarthritis, and neurological symptoms. HA20 develops at a younger age than BD and manifests itself by the presence of significantly more recurrent fevers, gastrointestinal involvement, and less common eye involvement than what is seen in BD [28]. Additionally, autoimmune diseases in patients with HA20 are unlike those seen in typical BD. The disease severity of HA20 varies from almost asymptomatic to severe regardless of the genotypes [26]. Treatment has not been established, but the use of corticosteroids, colchicine, tumor necrosis factor (TNF)-α inhibitors, and anakinra has been reported. Intractable cases have been treated with Janus kinase (JAK) inhibitors or hematopoietic stem cell transplantation (HSCT).

### 2.2. NF-κB Essential Modulator (NEMO, also Called IKBKG)

*NEMO* (OMIM: 300248) is located on chromosome Xq28 and encodes the regulatory gamma subunit of the IκB kinase (IKK) complex. This complex is involved in the proteolytic degradation of the IκB protein, which binds to NF-κB and inhibits its function, thereby enabling translocation of NF-κB into the nucleus and cytokine-associated gene transcription.

In males, the hypomorphic *NEMO* mutation causes X-linked anhidrotic ectodermal dysplasia with immunodeficiency (XL-EDA-ID; OMIM: 300291) [31]. XL-EDA-ID is characterized by severe, recurrent immunodeficiency-related infections in early infancy (or in the first years of life), ectodermal dysplasia, lymphedema, osteopetrosis, and early-onset inflammatory bowel disease. The highly variable phenotype is likely caused by different hypomorphic mutations, and intravenous immunoglobulin and prophylactics are used for its treatment. HSCT has been attempted in some cases.

In females, null or hypomorphic *NEMO* mutations cause incontinentia pigmenti (IP; OMIM: 308300) [32]. IP, an X-linked dominant disorder, is usually prenatally lethal in males. IP causes highly variable abnormalities of the skin, hair, nails, teeth, eyes, and central nervous system. Cells expressing the mutated X chromosome are eliminated selectively around the time of birth, so females with IP exhibit extremely skewed X-inactivation and heterogeneous clinical manifestations. With no specific treatment, symptomatic treatment is provided according to each symptom.

Additionally, NEMO deleted exon5-autoinflammatory syndrome (NEMO-NDAS), which is caused by splice-site variants, has been reported [33]. Patients with NEMO-NDAS show systemic inflammation including panniculitis rather than severe immunodeficiency.

Within 10 families, 13 female cases have been reported to exhibit pediatric-onset BD manifestations associated with IP or carry the *NEMO* mutation without IP [19,20,21]. *NEMO* gene analysis was performed in six subjects from three families and three *NEMO* mutations were identified (c.1217A > T/p.D406V, c.1191T > A/p.C397T*, c.613C > T/p.Q205*). Extremely skewed X-inactivation was found with the c.613C > T/p.Q205* mutation, but not with c.1217A > T/p.D406V. In the reported cases, oral and genital ulcers and gastrointestinal involvement were common, ophthalmologic involvement was low, but neurologic involvement was never reported. Treatments include corticosteroids, colchicine, and chlorambucil, and a TNF inhibitor with corticosteroid and colchicine was effective in one case of disease recurrence.

### 2.3. RELA

*RELA* (OMIM: 164014) encodes the RelA (p65) NF-κB subunit. The p50 (NFKB1)/p65 (RELA) heterodimer is the most abundant form of NF-κB. A 2-generation family (4 cases) carrying a heterozygous mutation in *RELA* (c.599+1G > A) were reported to present in their first years of life with chronic mucocutaneous ulceration and colitis (OMIM: 618287) [17]. TNF inhibitor treatment was effective in the proband. More recently, a heterozygous mutation in *RELA* (c.1459delC, p.H487fs), which causes RelA haploinsufficiency, was identified in a 3-generation family (5 cases) [18]. The loss-of-function mutation in *RELA* causes deficiencies in NF-κB signaling and leads to enhanced TNF-induced apoptosis. The authors reported that two cases of mucocutaneous ulceration and neuromyelitis optica were diagnosable as BD according to the ISGBD criteria. Both of the patients diagnosed with BD were successfully treated with a TNF inhibitor and the patient with neuromyelitis optica was successfully treated with an anti-CD20 antibody.

### 2.4. NFKB1

*NFKB1* (OMIM: 164011) and *NFKB2* (OMIM: 164012) encode NF-κB p50/p105 and p52/p100 subunits, respectively. *NFKB2* was first reported as the gene responsible for autosomal dominant (AD) common variable immunodeficiency (CVID) (OMIM: 615577), and this discovery was followed by *NFKB1* as another gene responsible for AD-CVID [34]. CVID is caused by heterozygous mutation in the *NFKB2* gene and is characterized by childhood-onset recurrent infections, asthma, and autoimmune features including alopecia areata and central adrenal insufficiency. In contrast, CVID caused by heterozygous mutation in the *NFKB1* gene (OMIM: 616576) has a highly variable age of onset and disease severity and is characterized by recurrent sinopulmonary infections, chronic obstructive pulmonary disease, and autoimmune features.

It has been shown that the −94ins/del in the ATTG promoter polymorphism of *NFKB1* is strongly associated with an enhanced risk of BD, especially in patients with ocular involvement [35]. It has also been reported that *NFKB1* mutation (c.667A < G, p.H67R) in one family (containing six cases) led to BD-like features and antibody deficiency [22]. The authors showed that the p.H67R variant reduced nuclear entry of p50 and decreased the transcriptional activity. Patients with the p.H67R variant had aphthae, gastrointestinal disease, febrile attacks, and the small-vessel vasculitis characteristic of BD, along with antibody deficiency.

### 2.5. TNF Receptor Superfamily Member 1A (TNFRSF1A)

*TNFRSF1A* (OMIM: 191190) encodes a type 1 TNF receptor called TNFR1. NF-κB is signal transductionally activated when TNF-α binds to TNFR1. It was reported in 1999 that *TNFRSF1A* mutations cause TNF receptor-associated periodic syndrome (TRAPS, OMIM: 142680) [36]. TRAPS is characterized by recurrent fever (often lasting beyond 1 week) with localized myalgia, painful erythema, arthralgia of the large joints, abdominal pain, conjunctivitis, and periorbital edema. Etanercept and anakinra corticosteroids are reportedly efficacious.

A patient with a heterozygous variant in *TNFRSF1A* (c.794A > G, p.K265R), which is considered a variant of uncertain clinical significance, presented with severe mucocutaneous ulcerations and pyrexia [12] after presenting with severe recurrent oral ulceration from 1 year of age. One episode required hospital admission for a large ulcer involving his entire hard palate. He also had punched-out skin ulcerations, lymphadenopathy, and pyrexia. While oral corticosteroid and colchicine were only partially effective in controlling his symptoms, considerable improvement was achieved when anakinra was initiated post-diagnosis.

## 3. Other Genes

### 3.1. DNA Ligase 4 (LIG4)

*LIG4* (OMIM: 601837) encodes a key component of the ubiquitous non-homologous end-joining (NHEJ) pathway. This pathway is essential for the DNA double-strand break (DSB) repair mechanism that is also utilized for the production of T and B lymphocyte receptors. LIG4 syndrome (OMIM: 606593) is caused by the homozygous or compound heterozygous mutation in *LIG4* first reported in 1990 [37]. LIG4 syndrome is characterized by combined immunodeficiency (CID), microcephaly, abnormal facial features, ionizing radiation sensitivity, growth failure, and susceptibility to malignancy. Initial treatment is supportive care, and HSCT is a curative treatment for CID and severe CID immunophenotypes.

A homozygous mutation in *LIG4* (c.2612G > A, p.R871H) was reported to mimic BD [16]. The male presented with recurrent meningitis episodes from age 7, and developed recurrent oral and genital ulceration, arthritis and anterior uveitis in addition to growth delay, abnormal facial features, and a dysembryoplastic neuroepithelial tumor. The patient was stable on colchicine and azathioprine but had intermittent orogenital ulceration flare-ups.

### 3.2. WDR1

*WDR1* (OMIM: 604734) encodes actin-interacting protein-1 (AIP1), which regulates cofilin-mediated actin depolymerization and disassembly. Periodic fevers, immunodeficiency and intermittent thrombocytopenia (PFIT, OMIM: 150550) is caused by homozygous or compound heterozygous mutation in *WDR1*. PFIT is characterized by recurrent early-onset respiratory infections, stomatitis, cutaneous infections, and autoimmune manifestations including chronic thrombocytopenia and anemia. Early death may occur, but HSCT may be curative.

Two sisters, each carrying a homozygous mutation in *WDR1* (c.887C > T, p.L293F) delivered from consanguineous parents, presented with BD-like symptoms initially [12]. Both had severe recurrent oral inflammation, which caused scarring and acquired microstomia in the younger sister, and recurrent perianal ulceration. Fever, arthritis, uveitis, upper respiratory tract infections, thrombocytopenia, and pyoderma gangrenosum-like rashes were also present. The elder sister initially responded well to anakinra but died from sterile systemic inflammation and multiorgan failure, whereas the other sister underwent successful HSCT.

### 3.3. Neutrophil Cytosolic Factor 1 (NCF1)

*NCF1* (OMIM: 608512) is located on chromosome 7q11 and encodes the p47^phox^ protein, a component of the NADPH oxidase complex. Homozygous or compound heterozygous mutations in *NCF1* cause autosomal recessive chronic granulomatous disease (CGD) (OMIM: 233700). The NADPH oxidase abnormality reduces the ability to produce active oxygen, and reduces the bactericidal ability of phagocytic cells such that the patient is susceptible to bacterial and fungal infections. Nevertheless, excessive inflammation such as granulomatous formation and refractory enteritis may occur. Treatment is mainly antibiotics, but HSCT should be considered in severe cases.

Three female CGD cases where BD was initially diagnosed have been reported [12,23,24], and the genetic analysis performed on two of these cases identified mutations in *NCF1*. All the cases presented initially with features compatible with a BD mucocutaneous disease type, and two of them developed more typical features of CGD with severe abscesses. Gastrointestinal inflammation was also observed in two of them.

### 3.4. Signal Transducer and Activator of Transcription (STAT) 1

STAT1, which is encoded by *STAT1* (OMIM: 600555), is an essential component of the extensive signaling pathway downstream of cytokine receptors, the JAK-STAT pathway, and is critical for both type I IFN (IFN-α/β) and type II IFN (IFN-γ) signal transduction. Immunodeficiency-31C (OMIM: 614162) is caused by autosomal dominant gain-of-function mutations in *STAT1*, as reported in 2011 [38]. Its common clinical manifestations are chronic mucocutaneous candidiasis in infancy or early childhood. Recurrent bacterial, viral, fungal, and mycoplasma infections, enteropathy, and autoimmune disorders such as hypothyroidism or diabetes mellitus may also be present. Immunodeficiency-31A (OMIM: 614892) and immunodeficiency-31B (OMIM: 613796) are both caused by loss-of-function mutation in STAT1. Immunodeficiency-31A is an autosomal dominant disorder that selectively affects the IFN-γ pathway, but not the IFN-α/IFN-β pathway, and causes a predisposition to mycobacterial infections. Contrastingly, immunodeficiency-31B is an autosomal recessive disorder affecting both the IFN-α/IFN-β and the IFN-γ pathways and therefore often causes severe and lethal mycobacterial and various other viral infections.

A gain-of-function mutation in *STAT1* (c.839T > G, p.L280W) has been reported to mimic mucocutaneous BD [12]. The symptoms in this case began at 7 months of age with non-infective oral and genital ulcerative lesions, recurrent fever, and colchicine, corticosteroids, azathioprine and adalimumab were initiated. However, severe oral and esophageal candidiasis emerged as well as recurrent chest infections and acute adrenocortical insufficiency. After diagnosis, treatment with a JAK inhibitor was under consideration.

### 3.5. Adenosine Deaminase (ADA) 2 (CECR1)

ADA2 adenosine deaminase catalyzes the deamination of adenosine and 2-prime-deoxyadenosine to inosine and deoxyinosine, respectively. Through their actions, ADAs deactivate extracellular adenosine and terminate signaling through adenosine receptors. ADA2 is secreted into the extracellular space, is highly expressed in myeloid cells, and is produced by activated monocytes, macrophages, and dendritic cells. *ADA2* (OMIM: 607575) mutations were first described in 2014 [39,40] and identified in patients with polyarteritis nodosa [39] and in patients with recurrent strokes [40]. Homozygous or compound heterozygous mutations in *ADA2* cause deficiency of ADA2 (DADA2, OMIM: 615688). DADA2 has been linked with an imbalance in the differentiation of monocytes towards proinflammatory M1 macrophages, and is characterized by systemic vascular inflammatory disorder with skin ulceration and recurrent strokes affecting small vessels in the brain and resulting in neurologic dysfunction. Recurrent fever, myalgia, lesions resembling polyarteritis nodosa, livedo, immunodeficiency and hematological manifestations are also described. There may be some similarities in the presentation of cutaneous, neurological, and vasculitis features between DADA2 and BD. The first-line treatment consists of TNF-α inhibitors, and HSCT has been successful in a group of patients presenting with hematological manifestations.

A homozygous mutation in *ADA2* (c.973-2A > G) has been described in two families with cases of BD-like manifestations [25]. One of two sisters presented with cranial nerve palsy at the age of 3, and developed genital ulceration, arthralgia, and fever. Her sister presented with erythema nodosum, recurrent oral ulceration, folliculitis, and arthralgia at the age of 10. After diagnosing DADA2, their treatment was switched to TNF-α inhibitors. Another case had high-spiking fever, erythema nodosum, arthralgia, severe aphthous stomatitis and ulcerative lesion in his gut.

### 3.6. Mediterranean Fever (MEFV)

*MEFV* (OMIM: 608107) encodes pyrin whose function as an innate immune sensor can trigger inflammasome formation, allowing the production of inflammatory mediators during infection. Familial Mediterranean fever (FMF) is caused by homozygous or compound heterozygous mutations (OMIM: 249100) and by heterozygous mutations (OMIM: 134610) in *MEFV*; these mutations were identified by the International and French FMF consortium who in 1997 identified them in a 115-kb FMF candidate interval on chromosome 16p [41,42]. FMF is characterized by recurrent attacks of fever and inflammation in the peritoneum, synovium, or pleura, accompanied by pain. Infrequently, erysipelas-like skin eruptions, meningitis, and amyloidosis may be present.

FMF and BD, which are both associated with neutrophilic inflammation, share several common characteristics in their symptoms, geographic distribution, and therapeutic responsiveness to colchicine. There are multiple case reports in which *MEFV* mutations were found in BD patients [8,12]. An association between *MEFV* and neuro-BD was also reported [43].

### 3.7. AP1S3

Adaptor protein complex 1 (AP-1), an evolutionarily conserved heterotetramer, promotes vesicular trafficking between the trans-Golgi network and endosomes. AP1S3, which is encoded by *AP1S3* (OMIM: 615781), is a core AP-1 subunit that is predicted to stabilize AP-1 heterotetramers. Heterozygous missense mutations in *AP1S3* were identified in patients with pustular psoriasis (2014) (OMIM: 616106) [44]. Generalized pustular psoriasis is a life-threatening disease characterized by sudden, repeated episodes of high-grade fever, generalized rash, and disseminated pustules. Immunosuppressive agents such as corticosteroids, cyclosporine, and TNF-α inhibitors are used to treat pustular psoriasis.

A case where a heterozygous mutation in *AP1S3* (c.11T > G, p.F4C) presented with BD-like symptoms has been reported [12]. She experienced recurrent fevers, mouth ulcers, facial and lip swelling, pustular skin lesions, myalgia and arthralgia, and had a good response to TNF-α inhibitor treatment.

### 3.8. LYN

*LYN* (OMIM: 165120) encodes the Src-family tyrosine kinase LYN, which regulates B-cell proliferation and antibody production in B cells via B cell receptor (BCR) signaling. LYN functions as an activator or inhibitor of phosphorylation downstream of BCR activation [45]. Lyn also physically interacts IRF 5 to inhibit ubiquitination and phosphorylation of IRF5 in the TLR-MyD88 pathway, thereby suppressing the transcriptional activity of IRF5 [46]. A genetic association between *LYN* and systemic lupus erythematous was found in mice and humans. In addition, *LYN*-associated AID caused by a mutation in *LYN* was reported to be associated with early-onset autoinflammatory phenotypes such as hepatosplenomegaly, purpuric skin rashes, periorbital erythema, testicular swelling, and positive autoantibodies [47]. A tyrosine kinase inhibitor was initiated and clinical improvement was observed.

A case with a heterozygous mutation in *LYN* (c.1523A > T, p.Y508F) presented with BD-like phenotypes [12] and a history of recurrent panniculitis, abdominal pain, mouth ulcers, and testicular pain associated with fever.

### 3.9. Trisomy 8

Constitutional trisomy 8 mosaicism is associated with mental retardation and multiple congenital abnormalities including joint contractures, deep palmar and plantar creases, corpus callosum agenesis, and skeletal and renal anomalies. Constitutional trisomy 8 mosaicism and acquired trisomy 8 are known to associated with various hematopoietic malignancies. A relationship has also been reported between BD-like diseases and myelodysplastic syndrome (MDS) with trisomy 8 [29]. It was reported that patients with BD and MDS experience more fever episodes and intestinal lesions than patients with BD without MDS, and trisomy 8 was found to be common (81.3%) in patients with BD and MDS [30]. Thus, it has recently been named trisomy 8-associated autoinflammatory disease (TRIAD) [48].

## 4. Conclusions

We have summarized the genes reported to be associated with BD. It is becoming clear that PID-related genes are important causes BD and BD-like phenotypes. Treatment may vary depending on the disease, so analyzing the patient’s genetic background is very important. It is anticipated that comprehensive gene analysis will help disentangle the etiology of BD.

## Figures and Tables

**Table 1:** Summary of PID-related genes and the chromosomes associated with BD-like phenotypes.

	Classification	Gene/Chromosome	Cytogenetic Location	Disease	Inheritance	Common Features	Reference Number *
**Classification of Inborn Errors of Immunity from the IUIS**	IDs affecting cellular and humoral immunity	*LIG4*	13q33.3	LIG4 deficiency	AR	Combined immunodeficiency, microcephaly, abnormal facial features, sensitivity to ionizing radiation, growth failure, susceptibility to malignancy	[16]
		*RELA*	11q13.1	RelA haploinsufficiency	AD	Chronic mucocutaneous ulceration	[17,18]
	Combined ID with associated or syndromic features	*NEMO* *(IKBKG)*	Xq28	EDA-ID	XLR	Recurrent severe infections caused by immunodeficiency, ectodermal dysplasia, lymphedema, osteopetrosis	[19,20,21]
				IP	XLD	Variable abnormalities of skin, hair, nails, teeth, eyes, and central nervous system	
				NEMO- NDAS	XLR/ XLD	Panniculitis, chorioretinitis, progressive B cell lymphopenia, hypogammaglobulinemia	
	Predominantly antibody deficiencies	*NFKB1*	4q24	NFKB1 deficiency	AD	Recurrent sinopulmonary infections, COPD, autoimmune features	[22]
	Congenital defects of phagocyte number or function	*WDR1*	4q16.1	PFIT	AR	Recurrent respiratory infections, stomatitis, cutaneous infections	[12]
		*NCF1*	7q11.23	CGD	AR	Recurrent infections, abscesses, lymphadenopathy, granulomatous formation, enteritis	[12,23,24]
	Defects in intrinsic and innate immunity	*STAT1*	2q32.2	ID 31A	AD LOF	Mycobacterial infections	[12]
				ID 31B	AR LOF	Severe mycobacterial and viral infections	
				ID 31C	AD GOF	Chronic mucocutaneous candidiasis, recurrent severe infections, enteropathy, autoimmune disorders	
	Autoinflammatory disorders	*ADA2*	22q11.1	DADA2	AR LOF	Skin ulceration, recurrent strokes affecting the small vessels of the brain, fever, polyarteritis nodosa	[25]
		*MEFV*	16p13.3	FMF	AR LOF/AD	Recurrent attacks of fever, inflammation in the peritoneum, synovium, or pleura, accompanied by pain	[12]
		*TNFRSF1A*	12p13.31	TRAPS	AD	Recurrent fever, localized myalgia, painful erythema, arthralgia, conjunctivitis, serositis	[12]
		*TNFAIP3*	6q23.3	HA20	AD LOF	Periodic fever, recurrent aphthous stomatitis, genital ulceration, intestinal symptoms, skin rash, polyarthritis, autoimmune disorders	[12,13,26,27,28]
		*AP1S3*	2q36.1	Pustular psoriasis	AR	High-grade fever, generalized rash, disseminated pustules	[12]
**Others**		*LYN*	8q12.1	*LYN*-associated AID	AD	Hepatosplenomegaly, purpuric skin rash, periorbital erythema, testicular swelling, positive autoantibodies	[12]
		chromosome 8		Trisomy 8		Mental retardation, joint contractures, deep palmar and plantar creases, corpus callosum agenesis, skeletal and renal anomalies	[29,30]

* Reference numbers for the cases with BD-like phenotypes; IUIS: International Union of Immunological Societies Expert Committee; EDA-ID: anhidrotic ectodermal dysplasia with immunodeficiency; IP: incontinentia pigmenti; NEMO-NDAS: NEMO deleted exon 5-autoinflammatory syndrome; PFIT: periodic fevers, immunodeficiency and intermittent thrombocytopenia; CGD: chronic granulomatous disease; ID: immunodeficiency; DADA2: deficiency of adenosine deaminase 2; FMF: familial Mediterranean fever; TRAPS: TNF receptor-associated periodic syndrome; HA20: A20 haploinsufficiency; AID: autoinflammatory disease; AR: autosomal recessive; AD: autosomal dominant; XLR: X-linked recessive; XLD: X-linked dominant; LOF: loss-of-function; GOF: gain-of-function; COPD: chronic obstructive pulmonary disease.

## Data Availability

Data sharing not applicable.
